# The genesis of an exceptionally lethal venom in the timber rattlesnake (*Crotalus horridus*) revealed through comparative venom-gland transcriptomics

**DOI:** 10.1186/1471-2164-14-394

**Published:** 2013-06-12

**Authors:** Darin R Rokyta, Kenneth P Wray, Mark J Margres

**Affiliations:** 1 Department of Biological Science, Florida State University, Tallahassee, FL 32306-4295, USA

## Abstract

**Background:**

Snake venoms generally show sequence and quantitative variation within and between species, but some rattlesnakes have undergone exceptionally rapid, dramatic shifts in the composition, lethality, and pharmacological effects of their venoms. Such shifts have occurred within species, most notably in Mojave (*Crotalus scutulatus*), South American (*C. durissus*), and timber (*C. horridus*) rattlesnakes, resulting in some populations with extremely potent, neurotoxic venoms without the hemorrhagic effects typical of rattlesnake bites.

**Results:**

To better understand the evolutionary changes that resulted in the potent venom of a population of *C. horridus* from northern Florida, we sequenced the venom-gland transcriptome of an animal from this population for comparison with the previously described transcriptome of the eastern diamondback rattlesnake (*C. adamanteus*), a congener with a more typical rattlesnake venom. Relative to the toxin transcription of *C. adamanteus*, which consisted primarily of snake-venom metalloproteinases, C-type lectins, snake-venom serine proteinases, and myotoxin-A, the toxin transcription of *C. horridus* was far simpler in composition and consisted almost entirely of snake-venom serine proteinases, phospholipases A_2_, and bradykinin-potentiating and C-type natriuretic peptides. *Crotalus horridus* lacked significant expression of the hemorrhagic snake-venom metalloproteinases and C-type lectins. Evolution of shared toxin families involved differential expansion and loss of toxin clades within each species and pronounced differences in the highly expressed toxin paralogs. Toxin genes showed significantly higher rates of nonsynonymous substitution than nontoxin genes. The expression patterns of nontoxin genes were conserved between species, despite the vast differences in toxin expression.

**Conclusions:**

Our results represent the first complete, sequence-based comparison between the venoms of closely related snake species and reveal in unprecedented detail the rapid evolution of snake venoms. We found that the difference in venom properties resulted from major changes in expression levels of toxin gene families, differential gene-family expansion and loss, changes in which paralogs within gene families were expressed at high levels, and higher nonsynonymous substitution rates in the toxin genes relative to nontoxins. These massive alterations in the genetics of the venom phenotype emphasize the evolutionary lability and flexibility of this ecologically critical trait.

## Background

Venomous snakes rely almost entirely on their complex, largely proteinaceous venoms for feeding and defense, resulting in strong selective pressures on the genes encoding venom components [1-4] and on the ultimate repositories of the venoms, the snakes’ prey [5] and predators [6]. Although molecular signals of positive selection have been repeatedly documented for individual venom components through sequence comparisons across species [1-4,7], such analyses characterize only minute portions of the full evolutionary stories of venoms. Proteomic approaches [8] can characterize full-venom patterns of divergence between species [9,10], but only in broad strokes, failing to differentiate members of large venom-gene families and to provide information on sequence divergence. Even the most complex venoms are simple in terms of the number of gene families or toxin classes present; the hundreds of proteins [11] typically belong to less than 20 gene families. Proteomic approaches therefore average out many of the details of venom evolution. Venom-gland transcriptomics [12-16] have the unrealized potential to combine many, but certainly not all, of the benefits of both approaches. With adequate sequencing effort, transcriptomes can provide the full-venom information of proteomics approaches as well as the information-dense gene sequences for molecular-evolutionary analyses [17], although post-transcriptional regulation could lead to significant discrepancies between venom content and expressed toxin mRNAs [18].

Snake-venom composition can vary significantly between species [18,19], within and between populations of a single species [18-25], and even ontogenetically within an individual [10,26-30]. This variation is related, at least in part, to differences in diets [31]. Some general, recurrent patterns have been identified within this extensive variation, including the type I/II rattlesnake-venom classification described by Mackessy [29], which emphasizes the inverse relationship between toxicity and metalloproteinase activity seen in many rattlesnake venoms. Snake-venom metalloproteinases (SVMPs) are enzymes that break down components of the capillary basement membrane, resulting in local and systemic hemorrhage. SVMPs are more generally known to disrupt hemostasis and cause inflammation and apoptosis [32]. Type I venoms have high metalloproteinase activity and high LD_50_ values (>1.0 *μ*g/g mouse body weight), whereas type II venoms have low metalloproteinase activity and low LD_50_ values (<1.0 *μ*g/g mouse body weight). High metalloproteinase activity and high toxicity appear to be incompatible properties of rattlesnake venoms [33]. Type I venoms are by far the most prevalent of the two venom types, appearing in 20 out of 26 rattlesnake taxa examined by Mackessy [29]. Mackessy [29] also revealed that different subspecies can have different venom types. For example, the massasauga (*Sistrurus catenatus*) expresses type I venom in some subspecies (*S. c. tergeminus*) and type II in others (*S. c. catenatus* and perhaps *S. c. edwardsi*). Other studies have shown ontogenetic shifts between venom types, with juveniles expressing type II venom but switching to type I as adults [34,35]. Some species, such as *C. durissus*, are known to express type II venom as both juveniles and adults, a pattern hypothesized to represent paedomorphism [10]. Despite the major differences in pharmacology and composition between these two venom types, evolutionary transitions between them appear to be common and to occur in parallel in several different species and perhaps even in different populations of the same species, despite the relatively short time (∼12.7 million years) since *Crotalus* diverged from *Sistrurus*[36]. The selective pressures favoring these transitions, the events triggering or enabling them, and the precise nature of any expression or genetic changes resulting in the altered venom properties are unknown. The determination of these unknowns not only has implications for our understanding of the evolution of major phenotypic innovations, but also has practical implications for snakebite treatment. Although type II venoms are the minority, bites from snakes with type II venoms show drastically different pathologies that might require unique treatment approaches.

The eastern diamondback rattlesnake (*C. adamanteus*) and the timber rattlesnake (*C. horridus*) are among the largest rattlesnake species, capable of reaching lengths of 2.4 m and 1.9 m, respectively [37]. *Crotalus horridus* occurs from New England and extreme southern Ontario, southward to northern Florida, and westward to eastern Texas and extreme southeastern Minnesota [38]. *Crotalus adamanteus* is a species of the southeastern coastal plain, ranging from extreme southeastern North Carolina, southward to the Florida Keys, and westward along the coast to extreme eastern Louisiana, and is also common on many of the Atlantic and Gulf barrier islands [38]. The two species are sympatric in parts of the Carolinas, Georgia, northern Florida, Alabama, Mississippi, and Louisiana, although they appear to be partitioned by habitat preference. *Crotalus adamanteus* is more often encountered in areas of high, dry, sandy soils, and *C. horridus* is more often found in low, wet bottomlands [37,39]. Both species are of conservation concern because of habitat loss and human persecution [40-43]. *Crotalus horridus* has been extirpated from many areas, particularly in the northern part of its range, and is classified as endangered in six states and threatened in five others. *Crotalus adamanteus* is listed as endangered in North Carolina and is currently being reviewed for federally threatened status under the Endangered Species Act. The diets of both species are similar, consisting primarily of rabbits, squirrels, rats, mice, and occasionally birds, with rabbits being more commonly consumed by the larger *C. adamanteus*[37]. *Crotalus horridus* also occasionally consumes frogs and snakes [37]. Despite their similarities in size and diet, the venoms from some populations of the two species show dramatic differences in pharmacological properties, composition, and toxicity. *Crotalus adamanteus* and most populations of *C. horridus* express type I venom [17,29], but at least two distinct, southern populations of *C. horridus* express venoms consistent with a type II classification [44].

Straight and Glenn [45] isolated an extremely lethal, heterodimeric phospholipase A_2_ (PLA2) presynaptic neurotoxin from *C. horridus*, which was homologous to Mojave toxin from *C. scutulatus*[46] and crotoxin from *C. durissus terrificus*[47]. Related toxins have also been found in *C. helleri*[48], *C. tigris*[49], neonates of *C. simus*[10], and rattlesnakes in the genus *Sistrurus*[50]. The *C. horridus* toxin was named canebrake toxin because it was discovered from a northern-Florida specimen belonging to the former subspecies *C. h. atricaudatus*, known colloquially as the canebrake rattlesnake. Glenn et al. [44] further characterized this neurotoxin, examined its geographic distribution, and found a complex pattern of venom composition in relation to the presence/absence of canebrake toxin and hemorrhagic activity. *Crotalus horridus* individuals fall into one of four venom types: type A venoms have canebrake toxin but no hemorrhagic activity, type B venoms lack canebrake toxin but have hemorrhagic activity, type A+B venoms have both canebrake toxin and hemorrhagic activity, and type C venoms have neither canebrake toxin nor hemorrhagic activity. Types A and B appear to be the most common types, suggesting a strong inverse relationship in venom composition between canebrake toxin and toxins such as SVMPs, which are major contributors to tissue damage and hemorrhage. Type B venom dominates throughout most of the range of *C. horridus* with only two known, disjunct regions where type A is common, one of which (southeastern South Carolina through eastern Georgia and northern Florida) falls in one of the regions of sympatry with *C. adamanteus*. Type A venom would be considered a type II rattlesnake venom under Mackessy’s classification, whereas type B would be a type I [29]. In terms of LD_50_ in mice, the order of decreasing toxicity for these venom types is: A > A+B > B > C. Analogous venom types, excluding type C, have been identified in *C. scutulatus*[46] and *C. helleri*[48] in relation to the presence or absence of Mojave toxin. These venom types, in particular types A and B, reflect vastly different prey incapacitation strategies and possibly different feeding ecologies, because types A and C lack predigestive effects. Low hemorrhagic activity could limit the maximum size of prey that can be consumed or prevent effective digestion at suboptimal temperatures, thereby inducing altitudinal, geographical, or seasonal limitations on foraging [33].

The venom-gland transcriptome of *C. adamanteus* from northern Florida has been extensively characterized by means of 454 pyrosequencing [4] and Illumina sequencing [17], and this species’ venom is clearly type I on the basis of its biochemical properties, LD_50_[29], and expressed venom genes [17]. The most abundant transcript in its venom gland encoded a myotoxin-A (i.e., crotamine; MYO), and SVMPs were the most abundant toxin class [17]. To compare venom-gland expression patterns between a rattlesnake with type I venom and one with type II and to elucidate the evolutionary genesis of these venom types, we sequenced the venom-gland transcriptome of *C. horridus* from northern Florida by means of Illumina technology, following the sequencing and *de novo* assembly approach used for *C. adamanteus*[17]. We provide the first comprehensive, sequence-based comparison of venoms between two closely related snake species and the first in-depth examination of toxin gene-family evolution, expression, and reorganization resulting from recent species divergence. We generated the first genome-scale analysis of snake molecular evolution on the basis of thousands of newly annotated nontoxins and compared the evolution of toxin sequences to these nontoxin sequences to determine whether toxins are unique in their evolutionary patterns. While a comparison between the venom-gland transcriptomes of the two venom types in *C. horridus* might have provided a more precise comparison of expression patterns underlying the two venom types, such a comparison would provide substantially less data on toxin and nontoxin molecular evolution and on patterns of gene-family evolution in snake venoms. By comparing the venom-gland transcriptomes of *C. horridus* and *C. adamanteus*, we provide the first transcriptome-based comparison between type I and type II rattlesnake venoms and the first genome-scale characterization of molecular divergence between two closely related venomous snake species.

## Results and discussion

### *Crotalus horridus* venom-gland transcriptome sequencing and assembly

We generated a total of 113,344,311 pairs of 100-nucleotide (nt) raw reads, and 104,457,593 pairs passed the Illumina quality filter. We merged 64,169,665 pairs into high-quality composite reads on the basis of their 3’ overlaps as described by Rokyta et al. [17] and Rodrigue et al. [51]. These composite reads had average lengths of 133 nt with average phred scores of 46 and were the only reads used for assembly. Although we could have simply aligned our *C. horridus* sequencing reads to the transcripts previously identified for *C. adamanteus*, we performed an independent *de novo* assembly to provide confirmation of the *C. adamanteus* annotations, increase the total number of gene sequences identified for the genus *Crotalus*, and avoid propagating any errors that might be present in the *C. adamanteus* assembly. We followed the iterative assembly approach of Rokyta et al. [17]. We began by running the Extender program described by Rokyta et al. [17] on a set of 1,000 random reads to identify any extremely high-abundance transcripts. This procedure resulted in 670 contigs, 386 of which had full-length coding sequences. After eliminating duplicates, we had 39 nontoxins and 27 toxins. The high number of duplicates was indicative of extremely biased expression in the venom glands. With these 66 transcripts, we filtered ∼37% of the original reads and performed a *de novo* assembly with NGen on 10 million of the unfiltered reads. This assembly produced 6,112 contigs from which we annotated 24 full-length toxin and 1,479 full-length nontoxin transcripts. These 1,503 transcripts were used to filter ∼30% of the previously unfiltered reads, and 10 million of the remaining reads were used in a second *de novo* NGen assembly. This assembly produced 7,084 contigs, and we annotated 25 full-length toxin and 1,080 full-length nontoxin transcripts from these contigs. These 1,105 transcripts were used in a third and final filtering step, removing ∼12% of the reads from the previous set, and 10 million of the unfiltered reads were used in a final *de novo* NGen assembly. Of the resulting 6,825 contigs, 16 were annotated as full-length toxins and 580 as full-length nontoxins. After eliminating duplicates, our procedure generated 3,031 unique, full-length nontoxin coding sequences and 61 unique, full-length putative-toxin coding sequences.

**Figure 1:**
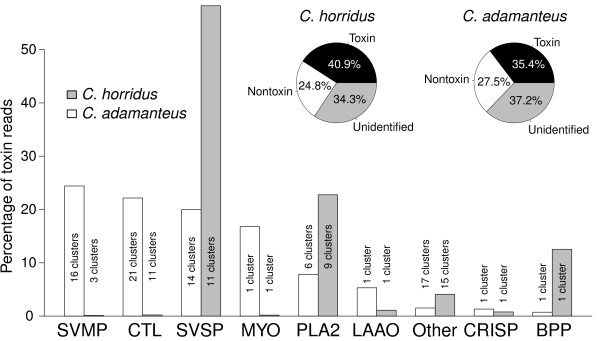
**The venom compositions of *Crotalus adamanteus * and *C. horridus * differed drastically, consistent with their respective type I and type II classifications.** The toxin-gene expression of *C. adamanteus* was dominated by snake-venom metalloproteinases (SVMPs), C-type lectins (CTLs), snake-venom serine proteinases (SVSPs), and a single, high-abundance myotoxin-A (MYO). These toxin classes, with the exception of SVSPs, were barely detectable in the venom-gland transcriptome of *C. horridus*, despite a similar number of reads mapping to toxins. The venom-gene expression of *C. horridus* was instead dominated by SVSPs, phospholipases A_2_ (PLA2s), and a single gene encoding bradykinin-potentiating and C-type natriuretic peptides (BPP). For both species, unique toxin sequences were grouped into clusters showing less than 1% nucleotide divergence. The other toxin-class abbreviations are as follows: cysteine-rich secretory protein, CRISP; and L-amino acid oxidase, LAAO.

Our 3,031 full-length, annotated nontoxin sequences accounted for 24.8% of the total sequencing reads, and our 61 toxin transcripts accounted for an additional 40.9%, illustrating clearly the extreme specialization of the venom gland. We were able to account for 65.7% of the sequencing reads (Figure 1). These percentages were similar to those of Rokyta et al. [17] who used the same overall sequencing and assembly approach for *C. adamanteus* (Figure 1). Our 3,092 annotated sequences (and the 3,002 sequences annotated for *C. adamanteus* by Rokyta et al. [17]) represent substantial fractions of the genes encoded by these rattlesnakes’ genomes; for comparison, 17,472 protein-coding genes were annotated from a full-genome assembly of the green anole lizard *Anolis carolinensis*[52]. The sequences generated in the present work and by Rokyta et al. [17] should facilitate genome sequencing and annotation for viperid snakes.

In all that follows, we used the percentage of reads mapping to a given transcript as a proxy for its expression level. This approach was used by Rokyta et al. [17] for *C. adamanteus*, against whose results we will be making extensive comparisons, and matches the measures used in the many Sanger-sequencing-based venom-gland transcriptomic studies for snakes [13,14,53-62]. A measure such as average coverage might help correct for any correlation between the number of reads mapping to a transcript and its length, but our data showed no significant relationship between these two values (*F*_1,3082_=0.60, *P*=0.44). The measures of reads per kilobase of exon model per million mapped reads (RPKM) [63,64] or fragments per kilobase of transcript per million mapped reads (FPKM) for paired-end reads [65] offer normalization for coding-RNA length and the total number of reads, providing an analog of the relative molar concentrations of transcripts. Note that our sequences, being processed mRNAs, lack introns, and this normalization for coding length is therefore less critical. More sophisticated normalization approaches have also been described [66] for RNA-seq data, but the optimal measure of transcript abundance remains under debate [67] and depends on the purpose and nature of the analyses. Generally, we were concerned with how transcriptional effort is apportioned among transcripts, which makes percentages a natural measure. In addition, percentages yield a form of compositional data [68], which has well-studied properties that enable natural comparisons among subsets. Such an approach is natural for gene-expression data [69].

### Type II *Crotalus horridus* toxin-gene expression patterns

Some of the 61 toxin sequences were similar enough to potentially confound estimates of their individual abundances, so for the purpose of estimating transcript abundances, we clustered these sequences whenever they showed less than 1% nt divergence in their coding sequences. The clustering threshold of 1% is arbitrary but matches the approach used by Rokyta et al. [17] for *C. adamanteus*. Our average composite-read lengths of 133 nt and a minimum nt divergence of 1% ensure that reads can generally be mapped uniquely to contigs. Clustering the original 61 toxin sequences resulted in 53 toxin clusters (Table 1). In all clusters, the mutations responsible for the differences between the sequences were at substantial frequencies (>10%) with high coverage, suggesting that these sequences were either alleles or recent duplicates rather than simply sequencing errors. Toxins were named by a toxin-class abbreviation, a number designating the cluster (if multiple clusters were present for the class), and a lower-case letter (if the cluster included more than a single sequence). Where necessary to differentiate between species, we preceded toxin names with “Cadam” to indicate those sequences identified in the *C. adamanteus* transcriptome and “Chorr” to indicate those sequences identified in the *C. horridus* transcriptome.

**Table 1:** **Expression levels of full-length toxin clusters for*Crotalus horridus***

**Rank**	**Cluster name**	**Cluster size**	**Length**	**% total reads**	**% toxin reads**
1	SVSP-1a	2	3,346	6.762	16.534
2	SVSP-3a	2	1,915	6.272	15.337
3	BPP-1a	2	1,484	5.120	12.519
4	SVSP-10	1	2,512	4.131	10.101
5	PLA2-2	1	880	3.346	8.183
6	SVSP-2a	2	2,089	3.305	8.082
7	PLA2-1a	2	933	2.884	7.051
8	PLA2-3	1	832	1.349	3.298
9	SVSP-7	1	2,209	0.900	2.200
10	SVSP-4a	2	2,354	0.827	2.021
11	VEGF-1	1	1,352	0.797	1.948
12	PLA2-4	1	1,303	0.687	1.681
13	PLA2-6	1	880	0.542	1.325
14	PLA2-9	1	812	0.502	1.228
15	SVSP-5	1	2,968	0.466	1.140
16	LAAO	1	2,890	0.452	1.106
17	SVSP-8	1	1,718	0.438	1.071
18	SVSP-11	1	2,704	0.384	0.940
19	CRISP	1	6,054	0.317	0.775
20	VESP	1	1,587	0.262	0.640
21	SVSP-9	1	1,341	0.189	0.461
22	SVSP-6	1	1,794	0.140	0.342
23	NGF	1	1,074	0.114	0.280
24	NUC	1	2,538	0.114	0.278
25	PDE	1	2,790	0.106	0.260
26	NF	1	1,230	0.088	0.215
27	MYO	1	415	0.080	0.195
28	VEGF-2	1	5,612	0.044	0.107
29	SVMPIII-1	1	3,395	0.040	0.099
30	KUN-1	1	2,246	0.040	0.099
31	HYAL	1	3,477	0.038	0.093
32	CTL-10	1	633	0.029	0.070
33	CREGF	1	1,625	0.028	0.069
34	GC	1	6,138	0.021	0.051
35	CTL-11	1	575	0.019	0.046
36	CTL-2a	2	669	0.011	0.027
37	KUN-2	1	4,317	0.008	0.020
38	CTL-1a	2	815	0.006	0.015
39	CTL-3	1	735	0.006	0.015
40	CTL-8	1	808	0.006	0.015
41	CTL-7	1	644	0.005	0.012
42	SVMPI	1	1,677	0.004	0.009
43	PDE-6	1	4,011	0.004	0.009
44	PDE-4	1	3,553	0.003	0.006
45	VF	1	5,139	0.002	0.005
46	CTL-4	1	697	0.002	0.004
47	CTL-5	1	743	0.002	0.004
48	PLA2-5	1	612	0.001	0.003
49	CTL-9	1	803	0.001	0.003
50	SVMPIII-2	1	2,499	0.001	0.003
51	CTL-6	1	637	0.001	0.002
52	PLA2-8	1	547	0.001	0.002
53	PLA2-7	1	546	0.001	0.001

**Figure 2:**
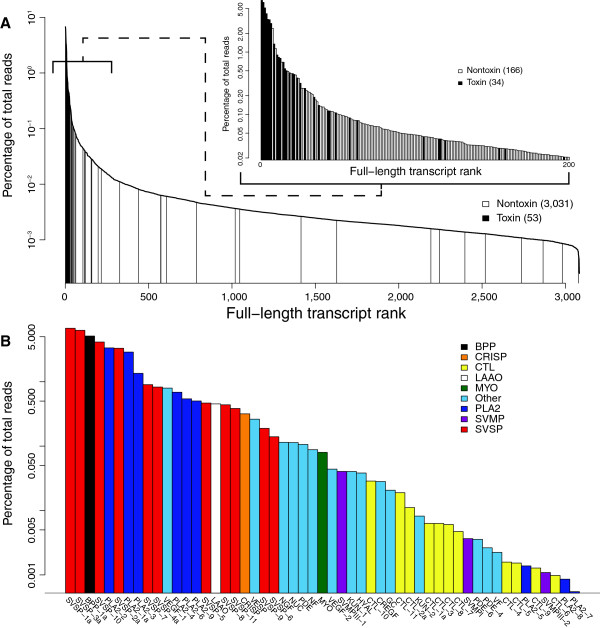
**The venom-gland transcriptome of *Crotalus horridus * was dominated by toxin transcripts.** The 61 unique toxin transcripts were grouped into 53 clusters with less than 1% nucleotide divergence for this analysis. Note that all vertical axes are on log scales. (**A**) The overall expression patterns with all of the full-length annotated sequences showed that toxins dominated the high-abundance transcripts. The inset shows a magnification of the 200 most-abundant transcripts. (**B**) Expression levels of the toxin sequences showed a clear dominance of snake-venom serine proteinases (SVSPs), phospholipases A_2_ (PLA2s), and a single gene encoding bradykinin-potentiating and C-type natriuretic peptides (BPP). Expression levels among members of toxin classes were highly variable. The remaining toxin-class abbreviations are as follows: C-type lectin, CTL; cysteine-rich secretory protein, CRISP; cysteine-rich with EGF-like domain, CREGF; glutaminyl-peptide cyclotransferase, GC; hyaluronidase, HYAL; Kunitz-type protease inhibitor, KUN; L-amino acid oxidase, LAAO; myotoxin-A, MYO; nerve growth factor, NGF; neurotrophic factor, NF; nucleotidase, NUC; phosphodiesterase, PDE; snake-venom metalloproteinase, SVMP; vascular endothelial growth factor, VEGF; venom factor, VF; and vespryn, VESP.

Toxin sequences were vastly overrepresented in the venom-gland transcriptome of *C. horridus* (Figure 2), accounting for 40.9% of the total reads (Figure 1). The seven most highly expressed genes were toxins, as were 15 of the 20 most highly expressed genes. Of the 200 most highly expressed genes, 34 were toxins (Figure 2A). As has been noted previously for other species [4,17,60], the venom gland of *C. horridus* is highly specialized for the production of toxic proteins.

**Figure 3:**
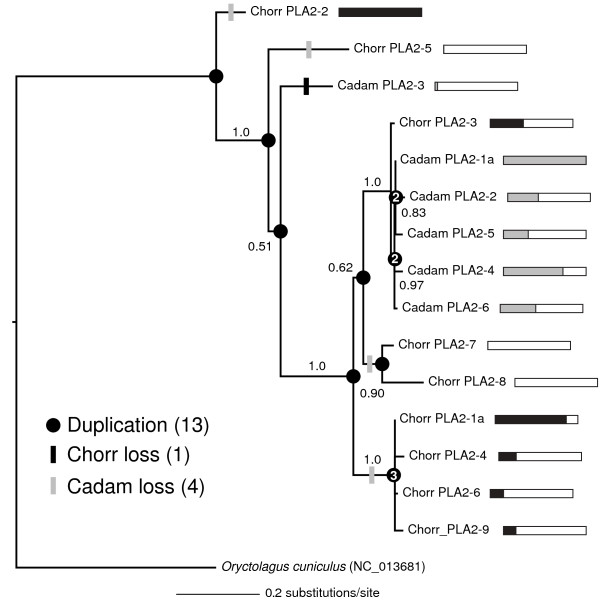
**Different clades were diversified and highly expressed in *Crotalus adamanteus * and *C. horridus * for the phospholipase A**_**2**_** (PLA2) gene family.** The bars adjacent to gene names give expression levels relative to the most highly expressed family member by species. A completely colored bar indicates the highest expression for the species. Clades comprising sequences from a single species either represent gene-family expansion for that species or gene-family contraction for the other. The four-gene clade including Chorr PLA2-1a and the five-gene clade including Cadam PLA2-1a are clear examples of this phenomenon. Duplication and gene-loss events were inferred by means of a duplication-loss parsimony model. We used a homologous nontoxin-PLA2 sequence from the european rabbit (*Oryctolagus cuniculus*) as an outgroup to root the phylogeny. Bayesian posterior probabilities are shown for clades for which the values exceeded 50%. Numbers within circles indicate that more than one duplication event was inferred for that node.

The venom of *C. horridus*, in terms of gene expression, was dominated by three classes of toxin: snake-venom serine proteinases (SVSPs), PLA2s, and bradykinin-potentiating and C-type natriuretic peptides (BPP; Figure 1 and Table 1). These three toxin classes accounted for 93.5% of the reads mapping to toxin sequences. The 11 clusters of SVSPs accounted for 58.2% of the toxin-encoding reads. These enzymatic venom components show highly variable substrate specificities but generally catalyze reactions that disrupt hemostatic mechanisms, including the coagulation cascade and the kallikrein-kinin, fibrinolytic, and complement systems [13,70]. The nine clusters of PLA2s accounted for 22.8% of the toxin reads. PLA2s show an astounding array of toxic functions, including neurotoxic, cardiotoxic, myotoxic, hemolytic, convulsive, anticoagulant, antiplatelet, edema-inducing, and tissue-damaging effects [71,72]. *Crotalus horridus* from the geographic region from which our animal was collected are known to express an extremely potent, heterodimeric, presynaptic *β*-neurotoxin known as canebrake toxin [44,45,73], which is homologous to the notoriously lethal Mojave toxin of *C. scutulatus* and crotoxin of *C. durissus terrificus*. These toxins comprise two noncovalently associated subunits, both of which are derived from PLA2 paralogs. The basic subunit is weakly toxic on its own and shows PLA2 activity. The acidic subunit is nontoxic alone and consists of three disulfide-linked polypeptide chains proteolytically derived from a PLA2-like precursor. The two most abundant PLA2 transcripts in the *C. horridus* venom-gland transcriptome, PLA2-2 and PLA2-1a, are most likely the basic and acidic chains of the canebrake toxin and account together for 15.2% of the toxin reads (Figure 2 and Table 1). PLA2-2 is 2.2% divergent at the nt level and 3.7% divergent at the amino-acid level from the crotoxin basic subunit (GenBank accession X12603). PLA2-1a is 3.2% divergent at the nt level and 8.4% divergent at the amino-acid level from the crotoxin acidic subunit (GenBank accession X12606). *Crotalus horridus* actually appeared to express four paralogous versions of the acidic subunit, including PLA2-1a, PLA2-4, PLA2-6, and PLA2-9 (Figure 3). The single BPP cluster in the *C. horridus* venom gland accounts for 12.5% of the toxin reads. These toxins are noted for lowering blood pressure in bite victims [13]. Together, these three major classes of toxins in high abundance suggest a unique viperid bite pathology consisting of coagulopathic, hypotensive, and neurotoxic effects.

Major classes typical of viperid venoms are notably at extremely low abundances in the *C. horridus* venom (Figure 1 and Table 1). We detected only three clusters of SVMPs, which accounted for just 0.11% of the toxin reads. These venom enzymes are responsible for most of the tissue damage and hemorrhage associated with most viperid bites [74] and contribute to predigestion of prey [33]. Similarly, the 11 C-type lectin (CTL) clusters accounted for just 0.22% of the toxin reads. These toxins, which typically function as multimers, are major components of hemorrhagic viperid venoms and contribute to the disruption of hemostasis by affecting plasma components and blood cells [75], ultimately leading to hemorrhage [76]. Finally, a single MYO cluster was detected, but it accounted for just 0.20% of the toxin reads. Although MYOs appear to be compatible with both type I and type II venoms [33], their primary role appears to be rapid prey incapacitation. A highly potent neurotoxin like canebrake toxin may render this particular toxin unnecessary.

We detected a number of additional low-abundance toxins in the venom-gland transcriptome of *C. horridus* (Figures 1 and 2 and Table 1). We identified an L-amino acid oxidase (LAAO) transcript, accounting for 1.11% of the toxin reads. LAAOs are associated with edema, apoptosis, and the inhibition of platelet aggregation [77]. A single cysteine-rich secretory protein (CRISP) sequence accounted for 0.78% of the toxin reads. CRISPs are thought to interfere with smooth-muscle contraction [78,79]. The remaining 15 putative toxin sequences accounted for 4.08% of the toxin reads and included two vascular endothelial growth factors (VEGFs), a vespryn (VESP) [80,81], a nerve growth factor (NGF), a nucleotidase (NUC), three phosphodiesterases (PDEs), a neurotrophic factor (NF), two Kunitz-type protease inhibitors (KUNs), a hyaluronidase (HYAL) [82], a cysteine-rich with EGF-like domain protein (CREGF), a glutaminyl-peptide cyclotransferase (GC) [83], and a venom factor (VF) [84,85].

### Type I versus type II: mRNA expression differences underlie rapid phenotypic evolution

The profiles of toxin classes expressed by type II *C. horridus* and its type I congener *C. adamanteus* were vastly different but consistent with their classifications as type I and type II venoms (Figure 1). These drastic differences were present despite the similarities in sizes, diets, and natural history of these two species and the similarity in the relative transcriptional effort expended on venom production (Figure 1). Type II venoms tend to be proteomically simpler than type I [49], and the transcriptome profiles that underlie these two venoms followed this trend. *Crotalus adamanteus* had 123 unique toxin sequences that fell into 78 clusters. *Crotalus horridus* had just 61 unique toxin sequences in 53 clusters. In terms of unique venom transcripts or toxin clusters, *C. horridus* had approximately 50 to 66% the complexity of *C. adamanteus*. In terms of major toxin classes, the simplicity of *C. horridus* venom was even more apparent. SVSPs, PLA2s, and, to a lesser extent, BPP made up most of the *C. horridus* venom transcripts (93.5% of the toxin reads), whereas the venom of *C. adamanteus* had a more even expression distribution over SVMPs, CTLs, SVSPs, MYO, PLA2s, and LAAO transcripts (Figure 1).

The prevalence of the more complex type I venoms in rattlesnakes [29] is difficult to reconcile with the advantages type II venoms appear to confer. The higher lethality of type II venoms implies greater efficacy in prey capture and reduced energetic costs, although toxicity has not been measured in natural, sympatric prey populations. Complex traits have been hypothesized to pay a cost in terms of their rates of adaptation [86], but this result depends on high levels of pleiotropy that do not appear to hold in most natural systems [87]. On the other hand, the higher complexity of type I venoms could lead to higher survival probability by means of functional redundancy, mutational robustness, and even increased rates of adaptation through an enlarged mutational target [88]. These potential advantages for type I venoms would provide more of a long-term evolutionary advantage, whereas the increased toxicity of type II venoms provide an immediate short-term fitness advantage. This hypothesized conflict between short-term and long-term advantages might explain both the overall prevalence of type I venoms among rattlesnakes and the fact that most species with type II venoms are still polymorphic for type I, even though type II venom dominates certain geographic regions.

Type II venoms are defined in part by their lack of hemorrhagic effects and, in particular, low SVMP activity. For the type I venom of *C. adamanteus*, SVMPs were the most highly expressed toxin class, accounting for 24.4% of the reads mapping to toxins. In stark contrast, SVMPs were almost undetectable in the expressed genes of the *C. horridus* venom-gland transcriptome, accounting for just 0.11% of the toxin reads (Figure 1). CTLs contribute to hemorrhage by either inhibiting or activating, and thereby depleting, coagulation factors [76]. They account for 22.2% of the toxin reads for *C. adamanteus* but just 0.2% of the toxin reads for *C. horridus* (Figure 1). The lack of hemorrhagic activity by *C. horridus* venom can therefore be explained by the lack of expression of genes responsible for this activity; we do not yet know whether all of these genes are still present in the genome but no longer expressed, or whether they have been lost from the genome. We do know, however, that some are present but expressed only in minute amounts (Figures 1 and 2).

Type II venoms are characterized by significant neurotoxic effects mediated generally by heterodimeric PLA2s homologous to crotoxin. The crotoxin homolog in *C. horridus*, canebrake toxin, is responsible for most of the toxicity of the type A venoms [44], and also accounts in part for the difference in PLA2 expression levels between *C. adamanteus* and *C. horridus* (Figure 1). *Crotalus adamanteus* expressed modest amounts of PLA2 transcripts (7.8% of its toxin reads), but, for *C. horridus*, PLA2s were the second most abundant class (22.8% of toxin reads). In *C. scutulatus*, which shows similar venom types to *C. horridus*, populations with predominantly type II venom show a corresponding absence of MYO [25], which causes myonecrosis and spastic hind-leg paralysis. The most abundantly expressed gene in the *C. adamanteus* venom-gland transcriptome was a MYO related to crotamine, but this gene was barely detectable in our type II *C. horridus*, accounting for just 0.2% of the toxin reads. This toxin’s probable role, prey incapacitation, is probably subsumed by the action of canebrake toxin in *C. horridus*.

Both species expressed high levels of SVSP transcripts, although SVSP transcripts accounted for a significantly larger portion of the toxin expression in *C. horridus* than in *C. adamanteus* (58.2% versus 20.0% of the toxin reads). Interestingly, the acidic subunit of crotoxin and its homologs are proteolytically cleaved into three peptides to produce the mature toxin; the protease responsible for the reaction is unknown, but could potentially be one or more serine proteinases, which might account for the higher expression of SVSPs in *C. horridus*.

A full proteomic characterization [8] and comparison will be necessary to determine whether the expression differences described above account for all of the differences in composition between the venoms of *C. adamanteus* and *C. horridus*. We have shown that dramatic changes in expression patterns for toxin gene classes underlie the correspondingly dramatic differences in venom composition and can account for the major pharmacological differences in the effects of the two venoms. In addition to the expression changes by toxin classes, changes of expression among paralogs within classes and sequence changes in individual toxins could also contribute to the different properties of the venoms (see below). Nonetheless, we have shown that extremely large and evolutionarily significant phenotypic changes between closely related species can be mediated by major changes in gene-expression patterns involving many genes, even over short evolutionary times. These dramatic changes in expression highlight a major advantage of chemical means of prey incapacitation and defense. Because venom genes, as far as is known, are expressed only in the venom glands (but see Casewell et al. [89]), major alterations in venom-gene expression can be achieved with no antagonistic pleiotropic effects. Similar large expression shifts for more typical genes would probably be strongly deleterious. Venoms are clean phenotypic modules that can undergo large changes with few, if any, deleterious pleiotropic effects, giving them potential for high evolvability.

### Type II venoms differ extensively among species

Although type II venoms are unified in their broad pharmacological properties, they are far from uniform in their compositions. In those cases where it has been investigated, the neurotoxicity of these venoms was derived from the heterodimeric PLA2 crotoxin and its homologs, but the few data available suggest differences in the remainder of the expressed genes as well as in the relative amount of crotoxin homologs. A low-coverage venom-gland transcriptome for *C. durissus collilineatus*[60], which expresses type II venom, showed that the transcripts encoding the two subunits of crotoxin account for 88% of the toxin-encoding transcripts. In contrast, PLA2 transcripts as a class only accounted for 22.8% of the toxin reads for *C. horridus*. SVSP transcripts were the most abundant toxin class for *C. horridus* at 58.2% of the toxin reads, but they only accounted for 2.5% of the toxin sequences for *C. durissus collilineatus*. Proteomic data from *C. simus* neonates [10] and *C. tigris*[49], both of which express type II venoms, suggest a closer agreement with our results. In both cases, however, the SVSPs were expressed at lower levels than for *C. horridus* (36.0% and 26.8% compared to 58.2%), and the PLA2s were expressed at higher levels (55.9% and 66.2% compared to 22.8%). Note, however, that we are comparing transcriptomes to proteomes, which do not always show quantitative agreement [90]. Nonetheless, these differences may be responsible for the lower LD_50_ for the venom of *C. tigris* (0.07 *μ*g/g) compared to type II *C. horridus* (0.22–1.0 *μ*g/g) [29,44].

If we assume that type I venom in adults is ancestral on the basis of its higher frequency among extant species [29], then the transition to type II venom has occured multiple times in rattlesnakes within the last ∼12.7 million years [36], a remarkable example of parallel phenotypic evolution. Calvete et al. [10] suggested that for *C. durissus*, type II venom represents a paedomorphic trait. If the ancestral state of rattlesnakes involved a switch between type II venom in neonates to type I as adults, such as is currently seen in *C. simus*[10], paedomorphism could provide a simple mechanism for frequent parallel evolution [33]. Unfortunately, the frequency of type II to type I ontogenetic shifts in rattlesnakes is unknown, although ontogenetic shifts in venom composition are not uncommon in viperids [10,26-28,91-93]. Of course, given the well-known among-species variation in venom composition of adult snakes, we expect similar levels of variation among juvenile venoms, thereby compounding the difficulties in elucidating the evolutionary history and patterns for snake venoms. A full investigation into the mechanisms of evolution of type II venom phenotypes in rattlesnakes, including determination of whether they represent paedomorphic traits, could provide insight into the repeatability of and constraints on large-scale phenotypic evolution.

### Toxin gene-family expansion and differential paralog expression

The evolution of animal-toxin multigene families is characterized by frequent gene gain and loss and strong positive selective pressures [1]. Such patterns have been described for PLA2s [2] and three-finger toxins [7] in snakes. Unfortunately, studies of these gene families rely on sparse and unsystematic sampling of toxin sequences within species and uneven sampling across species [7] because, until recently [4,17], complete, sequence-based characterizations of the venom components of a species were not feasible. Such sampling deficiencies probably introduce little, if any, bias into statistical tests of positive selection, but could have substantial impacts on the estimation of duplication and gene-loss rates. In particular, this bias could generate spurious signals for gene loss [7]. With our two high-coverage venom-gland transcriptomes for *C. horridus* and *C. adamanteus*, we provided the first detailed characterization of toxin gene-family evolution for snakes. Note that we could only detect sequences present in the genome and expressed in the venom glands, so gene loss in this context means that the gene was either deleted from the genome or it was no longer expressed. We only considered the evolution of the PLA2s and SVSPs, because these two families were the only two diverse gene families expressed at appreciable levels in both species.

**Figure 4:**
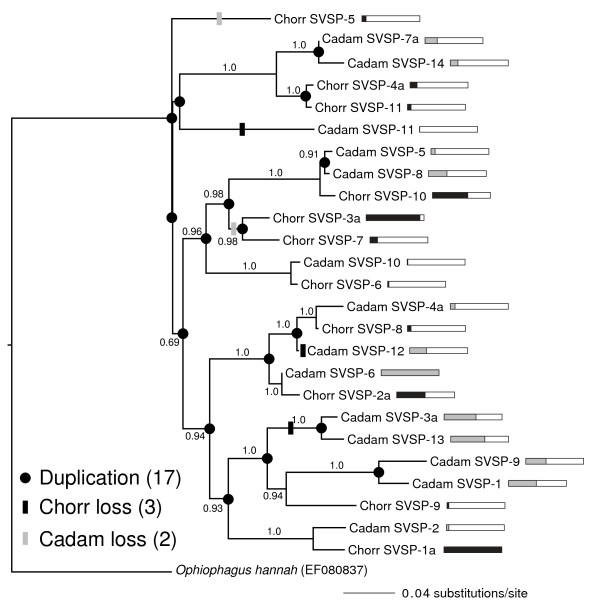
**The evolution of the snake-venom serine proteinase (SVSP) gene family in *Crotalus adamanteus * and *C. horridus * was characterized by duplication and changes in expression patterns.** The bars adjacent to gene names give expression levels relative to the most highly expressed family member by species. A completely colored bar indicates the highest expression for the species. Gene-family expansion and contraction appear to be less pronounced in SVSPs than phospholipases A_2_ (Figure 3). Duplication and gene-loss events were inferred by means of a duplication-loss parsimony model. We used a homologous SVSP from the king cobra (*Ophiophagus hannah*) as an outgroup to root the phylogeny. Bayesian posterior probabilities are shown for clades for which the values exceeded 50%.

We identified nine PLA2 transcripts for *C. horridus* and six for *C. adamanteus* (Figure 3) and used a related sequence from the european rabbit (*Oryctolagus cuniculus*) as our outgroup. This outgroup sequence was selected on the basis of tblastx searches of our PLA2 sequences against the NCBI nonredundant nt database, excluding results from viperids. To reconcile the PLA2 gene-family tree with the known species tree required at least 13 duplication events, one loss in *C. horridus*, and 4 losses in *C. adamanteus* (Figure 3). *Crotalus adamanteus* lacked orthologs for the two most highly expressed PLA2 paralogs for *C. horridus*: Chorr PLA2-1a and Chorr PLA2-2. Both species had bursts of species-specific gene-family expansion that involved multiple duplication events (Figure 3). The most highly expressed PLA2 for *C. adamanteus*, Cadam PLA2-1a, was part of a five-sequence clade unique to *C. adamanteus*. Similarly, the second most highly expressed PLA2 for *C. horridus*, Chorr PLA2-1a, was part of a four-sequence clade unique to *C. horridus*. These results suggest that gene-family expansion and expression levels are related, although this apparent relationship could simply reflect the use of expansion as a means of increasing expression levels. We identified 11 and 14 SVSP transcripts for *C. horridus* and *C. adamanteus*, respectively (Figure 4). The phylogeny of these sequences, with an elapid SVSP as an outgroup, showed a complex pattern of gene gain and loss, with an estimated 17 duplication events, three losses in *C. horridus*, and two losses in *C. adamanteus* (Figure 4). In constrast to the PLA2s, SVSPs showed no massive species-specific clade expansions, although species-specific duplication events were common (Figure 4). Similarly to the PLA2s, the SVSP clades expressed at high levels were different between the two species. For example, the most highly expressed SVSP for *C. horridus*, Chorr SVSP-1a, was paired with a low-expression *C. adamanteus* ortholog, Cadam SVSP-2. Our results provided the first detailed characterization of toxin gene-family evolution across closely related species. We found an overall pattern of gene-family expansion, with duplications greatly outnumbering losses. At the full-venom level, transcriptional effort among toxin classes was found to be dramatically different between *C. horridus* and *C. adamanteus* (Figure 1), and this same pattern repeated itself at a finer scale among paralogs within toxin classes (Figures 3 and 4). Even over the short time scale of divergence between congeners, expression levels for toxins were highly dynamic.

### Sequence divergence between *Crotalus adamanteus* and *C. horridus*

Claims abound of increased and exceptional evolutionary rates and selective pressures affecting snake venom genes [1-4], but these studies suffer from major limitations. With the exception of Gibbs and Rossiter [3], these studies average rates over the history of gene families and species and therefore capture only long-term patterns of molecular evolution. The most significant problem with these studies is the complete lack of a null expectation for molecular evolutionary patterns in snakes. We would like to know the proportion of venom genes that are evolving quickly over short time scales and whether these genes, and by implication the venom trait itself, are unique within the genome in terms of their evolutionary patterns. To address these questions, we used our annotated nontoxin sequences from *C. adamanteus* and *C. horridus* as the basis for our null expectation for molecular evolution and compared the patterns for toxins to the patterns for nontoxins. To generate our null expectations, we identified orthologous pairs of nontoxins for the two species by means of reciprocal-blast analyses. We excluded mitochondrially encoded sequences from these analyses because of their well-known high evolutionary rates. Each sequence for each species was searched against a database generated for the other species, and we performed separate searches on amino-acid sequences with blastp and nucleotide sequences with blastn. We only kept pairs of putative orthologs that were each others’ best matches for both analyses. From the 3,021 sequences from *C. horridus* and the 2,870 from *C. adamanteus*, we identified 1,903 putatively orthologous pairs. We excluded 90 pairs after alignment because their alignments contained more than 24 gapped positions, leaving 1,813 aligned pairs of orthologs. A similar treatment with the 79 toxin clusters from *C. adamanteus* and 53 from *C. horridus* yielded 30 toxin alignments.

**Figure 5:**
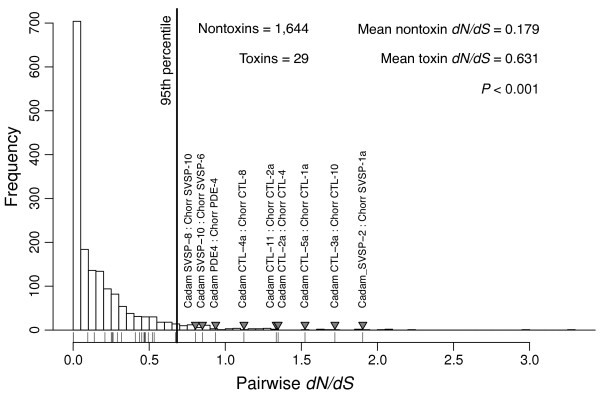
**The distribution of pairwise*dN/dS* ratios for toxins compared to nontoxins.** The histogram shows the null distribution of *dN*/*dS* for pairs of nontoxin orthologs identified by means of a reciprocal blast search. The vertical line denotes the cutoff for the 95th percentile for the nontoxins. The *dN*/*dS* values for the toxins are plotted below the histogram, and those toxin comparisons with values exceeding the 95th percentile for nontoxins are indictated with triangles and labeled with the names of the two sequences compared. We only expected 1.45 toxins to exceed this threshold if the distributions were the same for toxins and nontoxins. The *P* value is based on a Wilcoxon rank sum test and shows that the average *dN*/*dS* is significantly different between toxins and nontoxins. Comparisons with *dS*>0.1 were excluded. We also excluded comparisons with *dS*<0.001 to avoid inaccurately large *dN*/*dS* estimates.

**Figure 6:**
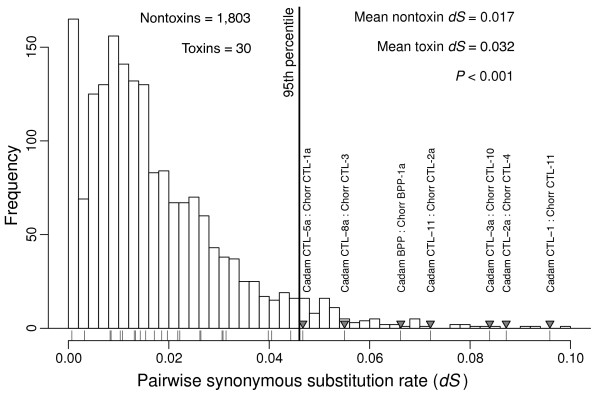
**The distribution of pairwise synonymous divergence (*dS*) for toxins compared to nontoxins.** The histogram shows the null distribution of *dS* for pairs of nontoxin orthologs identified by means of a reciprocal blast search. The vertical line denotes the cutoff for the 95th percentile for the nontoxins. The *dS* values for the toxins are plotted below the histogram, and those toxin comparisons with values exceeding the 95th percentile for nontoxins are indictated with triangles and labeled with the names of the two sequences compared. The *P* value is based on a Wilcoxon rank sum test and shows a significant difference between the average *dS* of toxins and nontoxins. Comparisons with *dS*>0.1 were excluded from the analysis. If the distributions were the same between toxins and nontoxins, we expected to see 1.5 toxins exceeding the threshold instead of the seven that were observed. Six of these seven toxin pairs were C-type lectins, a toxin class that showed a 100-fold reduction in expression in *C. horridus* relative to *C. adamanteus*. The CTLs above the threshold may represent a failure to find the true ortholog in the *C. horridus* transcriptome due to low coverage.

**Figure 7:**
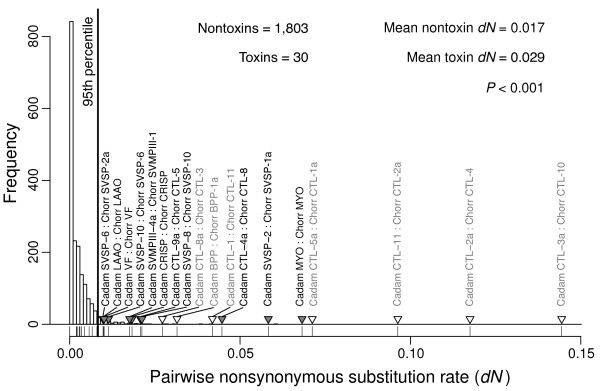
**The distribution of pairwise nonsynonymous divergence ( *dN *) for toxins compared to nontoxins showed an excess of outliers for toxins.** The histogram shows the null distribution of *dN* for pairs of nontoxin orthologs identified by means of a reciprocal blast search. The vertical line denotes the cutoff for the 95th percentile for the nontoxins. The *dN* values for the toxins are plotted below the histogram, and those toxin comparisons with values exceeding the 95th percentile for nontoxins are indictated with triangles and labeled with the names of the two sequences compared. The *P* value is based on a Wilcoxon rank sum test and shows a significant difference in the mean *dN* between toxins and nontoxins. Comparisons with *dS*>0.1 were excluded, and those comparisons that also exceeded the synonymous divergence threshold (Figure 7) are labeled with white triangles instead of grey. Excluding the *dS* outliers, we found that almost half of the toxin comparisons exceeded the threshold established by the nontoxins, rather than the expected 5%.

We conducted three separate analyses of molecular evolution. For the first (Figure 5), we compared the pairwise nonsynonymous to synonymous substitution-rate ratios (*dN*/*dS*) of the toxins to the nontoxins. Because our species were closely related and an accurate estimate of *dN*/*dS* requires sufficient synonymous divergence, we first estimated pairwise *dS* values and excluded pairs from the analysis if *dS*<0.001. In addition, we excluded pairs for which *dS*>0.1 to avoid spurious orthologs. These constraints left 1,644 nontoxin pairs and 29 toxin pairs. The average *dN*/*dS* for nontoxins was 0.18, and the average for toxins was significantly higher at 0.63 (*P*≪0.001 with a Wilcoxon rank sum test). As a class, toxins have a higher *dN*/*dS* than the background nontoxins. We used the individual values from the nontoxins to generate a null distribution for genomic pairwise *dN*/*dS* and compared our toxin values to this null distribution (Figure 5). Nine of 29 toxin pairs exceeded the 95th percentile of the null distribution, but we expected fewer than two exceedances if the distributions were the same. About 30% of toxin pairs have exceptionally high *dN*/*dS* ratios. As a class of sequences and at the individual level, toxins are distinct from nontoxins in terms of their *dN*/*dS* ratios. The low level of sequence divergence between our two species suggests that estimation of the *dN*/*dS* ratio may be inaccurate, so we conducted two additional analyses that were free of the issues of estimating a ratio where the denominator is expected to be quite small. We estimated null distributions of *dS* and *dN* separately for comparison with the corresponding values from the toxins (Figures 6 and 7). If toxins are uniquely under positive selection, we would expect to see higher rates of substitution for nonsynonymous mutations, but we have no reason to expect a corresponding increase in synonymous substitution rates. Synonymous substitution rate can therefore serve as a control. For both analyses, we excluded pairs with *dS*>0.1 as for our *dN*/*dS* analysis. For *dN*, we found that the average value for toxins was 0.029, which was significantly higher than the value of 0.017 for nontoxins (*P*≪0.001 with a Wilcoxon rank sum test). A majority of toxin pairs (18 of 30) exceeded the 95% threshold established by the nontoxin comparisons (Figure 7). However, we also found that toxins had a higher *dS* than nontoxins (*P*<0.001 with a Wilcoxon rank sum test), and seven of 30 toxin pairs exceeded the nontoxin 95% threshold. Six of these seven pairs consisted of CTLs, which were expressed at extremely low levels in the *C. horridus* transcriptome. These pairs possibly represent incorrectly paired paralogs that resulted from the low CTL coverage for *C. horridus*. Excluding these exceptional seven from the *dN* analysis still left 11 of 23 toxin pairs exceeding the *dN* threshold established by the nontoxins. As expected for two closely related species, the distributions of pairwise *dN* (Figure 7) and *dS* (Figure 6) showed peaks at zero, corresponding to pairs with little to no sequence divergence. The distribution for *dS* had a second mode corresponding to approximately 1% sequence divergence.

We have shown that even over the short amount of time since *C. horridus* and *C. adamanteus* shared a common ancestor, many, but by no means all, venom-encoding genes have evolved in an exceptional manner compared to other coding sequences in the genome. About 30% of toxin sequences showed evidence for a higher *dN*/*dS* ratio relative to the nontoxin background sequences, a pattern consistent with those sequences having experienced stronger and/or more prolonged positive selection. Only about 20% of toxins, however, showed *dN*/*dS*>1 (Figure 5), which is definitive and extremely conservative [94] evidence for positive selection as opposed to relaxed purifying selection. We also showed that about 50% of toxin sequences had exceptionally high nonsynonymous substitution rates relative to nontoxins, which is also consistent with strong, continual positive selection acting on toxins, although relaxed purifying selection cannot be ruled out. Our null distributions for these measures were derived from evolutionary patterns for a diverse array of nontoxin sequences that were expressed in the venom glands of both species and therefore may not reflect the prevailing patterns throughout the rest of the genome. Our large sample size of nearly 2,000 nontoxin genes, however, represents a substantial fraction of the coding sequences in the genome. In addition, the extremely different venom compositions of our two species resulted in a fairly small sample size for toxins. While this small sample size was sufficient to demonstrate the molecular-evolutionary distinctiveness of toxins compared to nontoxins, future comparisons among species with the same venom types will provide more power and higher-resolution characterizations of the differences in evolutionary patterns between toxins and nontoxins.

### Conserved nontoxin-expression patterns between *Crotalus adamanteus* and *C. horridus*

**Figure 8:**
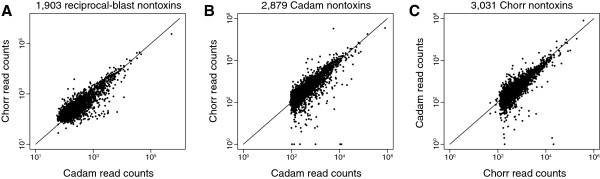
**Expression levels for nontoxins were conserved between *Crotalus adamanteus * and *C. horridus * despite major differences in toxin-expression patterns.** In all plots, the diagonal line indicates equal expression levels across species. (**A**) The comparison between species on the basis of orthologs identified by means of a reciprocal-blast search. (**B**) The comparison between species on the basis of aligning 10 million reads from each species against the nontoxin sequences from *C. adamanteus*. (**C**) The comparison between species on the basis of aligning 10 million reads from each species against the nontoxin sequences from *C. horridus*. Note that for (**B**) and (**C**), the distortion in the pattern at lower read counts is an artifact of the minimum-read-count threshold for annotating a contig.

The differences in expression patterns between *C. adamanteus* and *C. horridus* for genes encoding putative toxins are dramatic (Figure 1) and commensurate with the phenotypic difference in their venoms’ effects, but these toxin genes are only a minute portion of the genes expressed at high levels in the venom glands (Figure 2). Rokyta et al. [17] found that for *C. adamanteus*, the nontoxin expression was heavily biased towards genes involved in protein production and metabolism, as expected for a tissue specialized for protein secretion. Regardless of the particular proteins secreted, the basic protein-secretory function of snake venom-glands should be consistent across species, and we therefore expected expression patterns among nontoxin sequences to be more similar than patterns for toxins. To test this hypothesis, we conducted a reciprocal-blast analysis to identify orthologous sequences between the two species and compared their expression levels. For a pair of sequences to be included in our analysis, the two sequences had to have been each other’s best blast hit for both a nucleotide-based and an animo-acid-based search with blastn and blastp, respectively. We excluded mitochondrially encoded sequences for simplicity, leaving 2,870 nontoxins for *C. adamanteus* and 3,021 nontoxins for *C. horridus*. From these sequences, we identified 1,903 reciprocal blast matches (Figure 8). For expression levels, we used the number of reads mapping to each sequence based on aligning 10 million reads from each species to their own transcript sequences. Because the same number of reads were used for each species, this procedure is effectively equivalent to using a percentage. If the overall expression levels for nontoxins were similar between the two species, which appeared to be the case (Figure 1), and the expression levels of individual nontoxins were similar, we would expect a linear relationship between expression values for the two species with a slope of one and an intercept at zero. Instead, we found a good linear fit (*F*_1,1901_=3.3×10^4^, *P*≪10^−10^, *R*^2^=0.95), but with a slope of 0.48 and intercept of 417 reads when we used *C. horridus* expression values as a response variable. This lower-than-expected slope appeared to be caused by a single major outlier point representing a protein disulfide isomerase, the most highly expressed nontoxin identified for both species. Removal of this single point gave a good fit (*F*_1,1900_=1.4×10^4^, *P*≪10^−10^, *R*^2^=0.88) with a slope of 0.95 and an intercept of 51, indicating that expression levels for nontoxin sequences present for both species are generally in close agreement.

Our reciprocal-blast analysis indicated fairly extensive overlap between the nontoxins identified by means of independent *de novo* transcriptome assemblies for each species, but each species had ∼1,000 sequences without reciprocal-blast hits. This difference could reflect a difference in the identities of particular genes expressed in the two species’ venom-glands, which would represent a substantial difference in expression patterns, or it could simply represent a stochastic difference in the genes that were successfully assembled and annotated. To determine which of these two possibilities was true, we first filtered reads matching toxins for each species and then aligned 10 million of the filtered reads from each species against both sets of annotated nontoxin sequences. For the *C. adamanteus* nontoxins, *C. horridus* reads mapped to all but four template sequences (two more only had a single mapped read). For the *C. horridus* nontoxins, *C. adamanteus* reads mapped to all but a single nontoxin sequence (one more had only a single mapped read). The estimated expression levels in terms of number of mapped reads agreed well between both species for both sets of nontoxins (Figure 8). In addition, the total percentages of mapped reads were similar for both species for both sets of nontoxins. For the *C. adamanteus* transcripts, 42.6% and 41.2% of the reads mapped for *C. adamanteus* and *C. horridus*, respectively. For the *C. horridus* transcripts, 38.6% and 41.6% of the reads mapped for *C. adamanteus* and *C. horridus*, respectively. Note that in Figures 8A and 8B, read counts when mapping a species’ reads against its own transcripts showed a pattern of truncation on the lower end. This truncation reflects our procedure for selecting transcripts for annotation; we only tried to annotate contigs with at least 200 reads for each round of assembly.

Gene expression patterns generally appear to be under stabilizing selection [95-99], and the analyses above showed that nontoxin expression patterns were conserved across *C. adamanteus* and *C. horridus* despite major changes in expression patterns for toxin genes. This makes functional sense, because the same types of molecular machinery are needed to serve the secretory function of the venom-gland cells regardless of the particulars of the proteins being expressed, but it does raise questions about the regulatory control of gene expression in the venom glands. Given the large number of genes involved for both the toxins and nontoxins, and the relatively short divergence time between these species, it seems likely that toxins and nontoxins are under different regulatory control. This difference in control could contribute to the evolvability of venom by allowing large-scale changes in venom-gene expression without altering the underlying machinery for toxin production.

### Sequence accession numbers

The original, unmerged sequencing reads were submitted to the National Center for Biotechnology Information (NCBI) Sequence Read Archive under accession number SRA058913. The assembled and annotated sequences were submitted to NCBI as a Transcriptome Shotgun Assembly project. This Transcriptome Shotgun Assembly project has been deposited at DDBJ/EMBL/GenBank under the accession GAAZ00000000. The version described in this paper is the first version, GAAZ01000000.

## Conclusions

Rattlesnakes rely on their venoms for feeding and defense, and the ecological and evolutionary significance of these venoms ensures variation in their properties and compositions both within and between species. The most dramatic differences in rattlesnake venom properties correspond to a long-recognized dichotomy between neurotoxic and hemorrhagic venoms [29]. The timber rattlesnake (*C. horridus*) generally has hemorrhagic venom, but populations along the southern edge of their range express highly lethal, neurotoxic venom [44]. We sequenced the venom-gland transcriptome of an individual from one of these populations and compared the results to corresponding data from the eastern diamondback rattlesnake (*C. adamanteus*) [17], a congener with hemorrhagic venom.

The neurotoxic (type II) venom of *C. horridus* was about half as complex in terms of number of expressed genes as the hemorrhagic (type I) venom of *C. adamanteus*. This simplicity of type II venom might explain the general prevalence of type I venoms in rattlesnakes [29], despite the apparent advantage in potency of type II venoms. The higher complexity of type I venoms could provide evolutionary advantages in terms of functional redundancy, mutational robustness, and increased rates of adaptation through an enlarged mutational target. The primary basis for the lower complexity of the type II venom of *C. horridus* was the almost complete loss of expression of the two major classes of diverse hemorrhagic toxins in type I venoms, the CTLs and SVMPs. Overall, we found that the drastic difference in venom properties resulted from major changes in expression levels of toxin gene families, differential gene-family expansion and loss, changes in which paralogs within gene families were expressed at high levels, and higher *dN* and *dN*/*dS* values in the toxin genes relative to nontoxins. Despite the major expression differences for toxin transcripts, nontoxin expression patterns were consistent across the two species. Our work represents the first high-throughput comparative venom-gland transcriptomics study for snakes and therefore provides the first complete, in-depth look at patterns of toxin gene-family evolution, molecular evolution, and expression evolution in venomous snakes.

## Methods

### Venom-gland transcriptome sequencing

We followed the approach of Rokyta et al. [17] for the preparation and sequencing of the venom gland. We sequenced RNA from the venom glands of an adult female *C. horridus* from Bradford County, Florida. The animal weighed 1,134.7 g with a snout-to-vent length of 108 cm and a total length of 116 cm. We stimulated transcription in the glands by means of venom extraction under anesthesia [100]. The snake was anesthetized with a propofol injection (10 mg/kg) and exposure to isoflurane gas, and venom expulsion was initiated by means of electrostimulation. After allowing four days for transcription to be maximized [101], the animal was euthanized by injection of sodium pentobarbitol (100 mg/kg), and its venom glands were removed and transferred into RNAlater. The above techniques were approved by the Florida State University Institutional Animal Care and Use Committee (IACUC) under protocol #0924.

Sequencing and nonnormalized cDNA library preparation were performed by the HudsonAlpha Institute for Biotechnology Genomic Services Laboratory (http://www.hudsonalpha.org/gsl/). Transcriptome sequencing was performed essentially as described by Mortazavi et al. [63] in a modification of the standard Illumina methods described in detail in Bentley et al. [102]. Total RNA was reduced to poly-A+ RNA with oligo-dT beads. Two rounds of poly-A+ selection were performed. The purified mRNA was then subjected to a mild heat fragmentation followed by random priming for first-strand synthesis. Standard second-strand synthesis was followed by standard library preparation with the double-stranded cDNA as input material. This approach is similar to that of Illumina’s TruSeq RNA-seq library preparation kit. Sequencing was performed in one lane on the Illumina HiSeq 2000 with 100-base-pair paired-end reads.

### Transcriptome assembly

We followed the iterative transcriptome assembly approach of Rokyta et al. [17]. The majority of our read pairs had overlapping 3’ ends, so we merged these pairs into longer composite reads and updated their phred quality scores accordingly [17,51]. We also checked for and deleted any adapter sequences. Only these long, high-quality merged reads were used for assembly. We first eliminated extremely high-abundance transcripts by running the Extender program as a *de novo* assembler on a set of 1,000 random reads, as described by Rokyta et al. [17]. Full-length coding sequences were identified with blastx searches as described below. The resulting unique sequences were used as templates in a reference-based assembly with NGen3.1 with a 98% minimum match percentage. Ten million of the unassembled (i.e., unfiltered) reads were used in a *de novo* transcriptome assembly with NGen3.1 with the default settings for high-stringency, *de novo* transcriptome assembly for long Illumina reads, including default quality trimming. The high-stringency setting corresponded to a minimum match percentage of 93%, and we retained contigs comprising ≥200 reads. Any resulting contigs with full-length coding sequences were identified by means of blastx searches (see below). We performed two more iterative rounds of this filtering and *de novo* NGen assembly to yield the final set of contigs. We checked for duplicates by assembling all of the contigs with the SeqMan module of the DNAStar Lasergene software suite. We had several levels of quality control to prevent sequencing errors from being incorporated into our final sequences. We only used reads that passed Illumina’s quality filter and that were merged into overlapping, composite reads. All of our *de novo* assemblies with NGen used the default quality end trimming, and we only retained contigs with substantial coverage (≥200 reads).

We used blastx searches as implemented in mpiblast version 1.6.0 (http://www.mpiblast.org/) for identification and annotation of our contigs. Contig sequences were searched against the NCBI nonredundant protein database (nr) with an E-value cutoff of 10 ^−4^, and only the best 10 matches were retained. For toxin identification, hit descriptions were searched for a set of keywords based on known snake-venom toxins and protein classes; any sequence matching these keywords was checked for a full-length putative-toxin encoding sequence. The remaining contigs were screened for those whose match lengths were ≥90% of the length of at least one of their database matches. This step was intended to eliminate fragmented or partial sequences before attempting annotation. Each annotated sequence was checked and confirmed by hand in the SeqBuilder module of the DNAStar Lasergene software suite.

We estimated transcript abundances using high-stringency reference-based assemblies in NGen3.1 with a minimum match percentage of 95. Ten million of the merged reads were mapped onto the full-length, annotated transcripts, and the percentage of reads mapping to each transcript was used as a proxy for abundance. To compare nontoxin expression levels across species, we aligned each species’ reads against both their own and the other species’ annotated nontoxin transcripts using reference-based assembly in NGen3.1 with a minimum match percentage of 95. For each species, we used 10 million reads, after first filtering reads mapping to toxin contigs.

### Analysis of molecular-evolutionary patterns

Relationships among toxins within toxin families were determined by means of maximum-likelihood phylogeny estimation with PAUP*, version 4.0b10 [103] and the iterative search strategy described by Rokyta et al. [104]. All alignments were constructed with ClustalW [105]. Evolutionary models were selected using MrModelTest version 2 with Akaike Information Criterion values. Nodal support was estimated by means of posterior clade probabilities using MrBayes version 3.1.2 [106]. Markov chain Monte Carlo searches were run for 10 million generations with four chains, the temperature parameter set to 0.2, and samples taken every 1,000 generations. Samples from the first one million generations were discarded as burn-in. To infer duplication and loss events on the estimated phylogenies by reconciling them with the known three-species phylogenies, we used Notung 2.6 [107,108].

To compare molecular-evolutionary patterns of toxins to nontoxins, we identified orthologous pairs of sequences from our two species by means of a reciprocal-blast analysis. We constructed nucleotide and amino-acid sequence databases for each species, excluding mitochondrially encoded sequences, and blasted each sequence from each species against the database generated for the other species. We performed blastn and blastp searches for each sequence with an E-value cutoff of 10^−6^. For blastn searches, we used the entire sequence, including untranslated regions. Putatively orthologous pairs were only retained if the two constituent sequences were each other’s best matches for both the nucleotide-based and amino-acid-sequence-based analyses. The coding sequences of retained pairs were aligned using ClustalW [105]. Alignments with more than 24 gapped positions in the coding sequences were excluded from further consideration to avoid considering potentially incorrectly annotated sequences. For the remaining orthologous pairs, we estimated the pairwise synonymous (*dS*) and nonsynonymous (*dN*) substitution rates and the pairwise ratios of nonsynonymous to synonymous substitution rates (*dN*/*dS*) with codeml from PAML version 4.4 [109,110].

## Abbreviations

BPP: Bradykinin-potentiating and C-type natriuretic peptides; CTL: C-type lectin; CREGF: Cysteine-rich with EGF-like domain; CRISP: Cysteine-rich secretory protein; dN: Nonsynonymous substitution rate; dN/dS: Ratio of nonsynonymous to synonymous substitution rates; dS: Synonymous substitution rate; FPKM: Fragments per kilobase of transcript per million mapped reads; GC: Glutaminyl-peptide cyclotransferase; HYAL: Hyaluronidase; KUN: Kunitz-type protease inhibitor; LAAO: L-amino acid oxidase; MYO: Myotoxin-A (crotamine); NGF: Nerve growth factor; NF: Neurotrophic factor; nt: Nucleotide; NUC: Nucleotidase; PDE: Phosphodiesterase; PLA2: Phospholipase A2; RPKM: Reads per kilobase of exon model per million mapped reads; SVMP: Snake venom metalloproteinase; SVSP: Snake venom serine proteinase; VEGF: Vascular endothelial growth factor; VESP: Vespryn (ohanin-like)

## Competing interests

The authors declare that they have no competing interests.

## Authors’ contributions

The project was conceived and planned by DRR and KPW. DRR, MJM, and KPW collected and analyzed the data. DR and KPW wrote the manuscript. All authors read and approved the final manuscript.
